# Alleged Cure for Croup

**Published:** 1847-01

**Authors:** 


*Alleged Cure for Croup.*—In the American Water-Cure Advocate, a leading column is headed thus:—"Important Discovery to Parents."—which goes on to relate that Dr. Wigand, of Boston, has published a work, in which a discovery is announced that he cures the croup by cold water, exclusively. After a patient examination of the printed catalogue of Boston Physicians, and the Directory for 1846, the name of Wigand has not been found. Yet this name is passed round the ring in the country, for authority in a particular line of practice. If a book has actually been written by this celebrated croup curer, it has made no sensation here, however much it may have moved the waters in Ohio. The locality of the author may be in the neighborhood, perhaps, of the *Mesmeric College*, that was gravely asserted, last season, by distant newspapers, to have been organized in *Billerica street!* of which the wonderful and very surprising Dr. Dodd was both president and professor. This same Water-Cure Advocate speaks in raptures of the Green Mountain Spring, by D. Mack, as an ably conducted journal—which is certainly a discovery, since he is the only person who can speak thus and keep his countenance.—*Boston Med. and Surg. Jour.*

